# Silent Effects of the COVID-19 Pandemic: Parental Attachment in Pediatric Patients

**DOI:** 10.7759/cureus.70937

**Published:** 2024-10-06

**Authors:** Nazmi Mutlu Karakas, Selin Kuzucu

**Affiliations:** 1 Childhood Disease, Gazi University Medical School, Ankara, TUR; 2 Pediatric Medicine, Sorgun State Hospital, Yozgat, TUR

**Keywords:** adolescence, attachment, covid-19, covid-19 pandemic, parent-child relationship

## Abstract

Aim: The COVID-19 pandemic led to isolation measures intended to curb the spread of infection, which are believed to have negatively affected children's psychological well-being. This study examines the impact of the pandemic on parental attachment with children.

Method: A cross-sectional study was conducted with children visiting a general pediatrics clinic between April 1 and 20, 2023. Their COVID-19 infection history was recorded, and the Kern’s Attachment Questionnaire was used to assess parental attachment in children who had contracted COVID-19 and those who had not.

Results: The study involved 716 patients aged 9-12, divided into two groups: those who had contracted COVID-19 (n=253) and those who had not (n=463). Kern’s Attachment Questionnaire scores for mothers were 46.3 for children with COVID-19 and 49.4 for those without. For fathers, the scores were 43.9 and 48, respectively. Children who had contracted COVID-19 showed significantly lower attachment scores to both mothers and fathers compared to the control group (p=0.04, p=0.00).

Conclusion: The pandemic's long-term biopsychosocial effects are evident, with increased stress and negative experiences posing risks to child development, particularly in terms of parental attachment. While early attachment begins in infancy, it continues to evolve. This study underscores the need for behavioral and psychological follow-up for adolescents in the post-pandemic period.

## Introduction

The COVID-19 pandemic has become a global concern, impacting people worldwide [1]. To slow the spread of the virus and reduce transmission, many countries implemented social distancing and isolation measures [1]. Among these, home quarantines and school closures were the most common strategies [1,2]. In Turkey, COVID-19 patients were required to undergo a 14-day home isolation period [2]. While these measures were crucial in preventing infections, they also had adverse effects on the physical and psychological health of many individuals [3].

Pandemics or epidemics, like COVID-19, pose potential risks to child development due to factors such as the threat of illness, school closures, peer isolation, and increased parental stress [3,4]. Adolescence is a critical period for identity formation [5]. Adolescents, who have not yet achieved the psychological maturity to handle challenging situations like adults, face greater difficulties in maintaining their mental health during stressful periods such as a pandemic [5]. These stressful conditions are considered adverse childhood experiences and are associated with long-term consequences, including disruptions in brain development as well as cognitive, mental, and physical functions, due to toxic stress [4,6].

Exposure to stress factors can lead to cognitive, emotional, and physical fatigue in both children and adults, potentially increasing tension in parent-child relationships [6]. Attachment plays a crucial role in the relationship between parents and adolescents [7]. The attachment process stems from interactions with the parent or caregiver and influences various cognitive, emotional, and behavioral domains through self-perception, directly affecting an individual's biopsychosocial well-being [6,7].

Although it was initially believed that forced cohabitation due to COVID-19 isolation would strengthen family relationships, limitations on social interactions outside the family and other pandemic-related stressors have negatively impacted parent-child relationships [6,8,9].

This study aims to demonstrate that adolescents who contracted COVID-19 experienced lower levels of parental attachment compared to those who did not contract the virus.

## Materials and methods

This cross-sectional study was conducted using a face-to-face survey with patients who visited the General Pediatrics Outpatient Clinic at Gazi University Medical Faculty Hospital between April 1-30, 2023. The purpose and methodology of the study were first verbally explained to the patients and their parents. A total of 719 patients, whose written consent was obtained, were included in the study. The inclusion criteria required patients to be aged 9-12 years, without neuromotor developmental delays, and with compatible cognitive functions. Patients with neuromotor diseases or mental retardation were excluded as they would not have been able to answer the questions accurately.

The patients had undergone a 14-day home isolation period following asymptomatic or mild COVID-19 infections that did not require hospitalization. These patients remained in isolation with both parents and had not experienced parental loss, death, or a grieving process. Their history of COVID-19 infection was confirmed based on polymerase chain reaction (PCR) test results documented in their medical records.

It was planned to administer Kern's Attachment Questionnaire to the participating patients and to collect data on age, gender, and reason for admission to the outpatient clinic. Patients were also asked about their COVID-19 infection history, and their PCR test results were accessed through national health database records.

The Kern's Attachment Questionnaire was administered while the patient was alone in a quiet room, allowing them to respond objectively with awareness of their parents. The Kern's Attachment Questionnaire is designed to measure the level of secure attachment children have toward their parents. It consists of 15 items and is applicable to children aged 9-12. Each item presents two contradictory statements (e.g., "Some kids…but some kids…"). Participants are first asked to decide which of the two types of children described on either side of the "BUT" box is more similar to them. They are then asked to indicate how similar they feel to the chosen child.

The questionnaire is scored on a scale ranging from a minimum of 15 to a maximum of 60 points. A higher score indicates a stronger, more secure parental attachment. Kern's Attachment Questionnaire was adapted into Turkish by Nebi Sümer and Meltem Anafarta Şendağ in 2009 from the "Hope and Secure Attachment to Parents in Middle Childhood" scale [10]. The validity of the questionnaire was found to be 0.84 and 0.85, respectively, supporting its single-dimensional structure through analyses using Varimax rotation and internal consistency measures. Examination of correlations between the items revealed a strong relationship between attachment to parents (r=0.56, p<0.001). Additionally, as predicted, a significant relationship (with correlation values ranging from 0.32 to 0.50) was found between attachment to both parents and self-perceptions and anxiety in different domains. It has been reported that no permission is required for the use of the scale.

Data analysis was conducted using the SPSS Statistics version 11.5 software (SPSS Inc. Released 2002. SPSS for Windows, Version 11.5. Chicago, SPSS Inc.). Variables were classified as either categorical or numerical. Frequency distributions (count, percentage) and descriptive statistics (mean, standard deviation) were used to summarize the data. The Pearson chi-square test, Fisher's exact test, continuity correction chi-square test, Student's t-test, and Mann-Whitney U test were employed for inferential analyses. Statistical significance was set at p<0.05, with a Type I error threshold of less than 5%.

Ethics committee approval for the study was granted by the Institutional Review Board of Gazi University Medical School (approval number: 2023-219).

## Results

The study included 716 children, with a mean age of 10.4 (1.1) years. Of these, 253 children (35.3%) had contracted COVID-19, while 463 children (64.7%) had not. The female-to-male ratio was 1.08. The age distribution is provided in Table 1.

**Table 1:** Sociodemographic characteristics of the patients SD: standard deviation, COVID-19: coronavirus disease 2019

	n=716 (%)
Age (years)	
Mean (SD)	10.4 (1.1)
Median (min-max)	10 (9-12)
Age (years)	
9	178 (24.8)
10	191 (26.6)
11	174( 24.3)
12	173 (24.1)
Gender	
Female	372 (51.9)
Male	344 (48.1)
History of COVID-19 infection	
Yes	253 (35.3)
No	463 (64.7)

The most common complaints upon admission include fever, symptoms of upper respiratory tract infections, fatigue, abdominal pain with vomiting, suspected shortness of breath, and pre-surgery consent. Other complaints listed under the "Others" section include symptoms such as headaches, convulsions, skin rashes, and joint pain (Table 2).

**Table 2:** Application complaints of the patients

	n=716 (%)
Fever and upper respiratory tract infection	172 (24.0)
Dysorexia, fatigue	135 (18.7)
Stomach ache, vomiting	116 (16.2)
Suspicion of short stature	50 (6.9)
Consent before surgery	46( 6.2)
Others	200 (28.0)

When the Kern's Attachment Questionnaire scores of 716 patients were evaluated, the mother attachment score ranged from 21 to 60, with a mean score of 47.3 (7.7). The father attachment score ranged from 18 to 60, with a mean score of 45.5 (8.8) (Table 3).

**Table 3:** Kern's Attachment Questionnaire scores of all patients SD: standard deviation

Mother attachment score	
Mean (SD)	47.3 (7.7)
Median (min-max)	49 (21-60)
Father attachment score	
Mean (SD)	45.5 (8.8)
Median (min-max)	46 (18-60)

When the attachment scores of mothers and fathers were compared by gender, the differences were not statistically significant (p=0.72, p=0.22, respectively) (Table 4).

**Table 4:** Kern's Attachment Questionnaire scores of all patients by gender * Mann-Whitney U test was used to analyze the p-value. SD: standard deviation

	Female	Male	p-value
Mother attachment score			
Mean (SD)	47.3 (7.7)	47.7 (7.3)	0.72*
Median	48 (21-60)	48 (21-60)	
Father attachment score			
Mean (SD)	45 (8.9)	45,3 (8.2)	0.22*
Median	46 (21-60)	45 (24-60)	

Pearson correlation analysis was used to examine the relationship between the mother attachment score and the father attachment score. A positive correlation exists between the mother and father attachment scores, and this finding is statistically significant (p=0.00) (Table 5).

**Table 5:** Relationship between mother and father attachment scores * Correlation is significant at the p<0.01 level, Pearson correlation test

		Mother attachment score
Father attachment score	Pearson r	0.59
	p	<0.001*

The patients were compared based on their scores from Kern's Attachment Questionnaire and their COVID-19 infection status. Among 253 patients with COVID-19 infection, the average score for the mother dimension was 46.3 (7.7). In contrast, the average mother score for 463 patients without COVID-19 infection was 47.9 (8.8). The mother score from Kern's Attachment Questionnaire was lower in patients with COVID-19 infection, and this difference is statistically significant (p=0.03) (Table 6).

**Table 6:** Kern's Attachment Questionnaire score of mothers in terms of patients' COVID-19 infection status * Mann-Whitney U test was used to analyze the p-value COVID-19: coronavirus disease 2019, SD: standard deviation

	History of infection with COVID-19		
	Yes	No	p-value
Kern's Attachment Questionnaire score - mother			
Mean (SD)	46.3 (7.6)	47.9 (7.7)	0.03*
Median (min-max)	47 (21-60)	49 (21-60)	

The patients were compared based on their COVID-19 infection status using the Kerns Attachment Questionnaire scores. Among the 253 patients with COVID-19 infection, the average father score was 43.9 (8.8). In contrast, the average father score for the 463 patients without COVID-19 infection was 46.4 (8.7). The father score from the Kern's Attachment Questionnaire is lower in patients with COVID-19 infection, and this difference is statistically significant (p=0.000) (Table 7).

**Table 7:** Kern's Attachment Questionnaire score for fathers in terms of patients' COVID-19 infection status * Mann-Whitney U test was used to analyze the p-value. COVID-19: coronavirus disease 2019, SD: standard deviation

	History of infection with COVID-19		
	Yes	No	p-value
Kern's Attachment Questionnaire score - father			
Mean (SD)	43.9 (8.8)	46.4 (8.7)	<0.001*
Median (min-max)	43 (18-60)	48 (18-60)	

## Discussion

In our study, adolescents who contracted COVID-19 and underwent a period of isolation exhibited lower parental attachment scores. This finding may be associated with various factors [8,9,11,12]. During the pandemic, the time spent at home with family increased due to isolation measures [3,11,12]. Although it was initially believed that this would have positive effects on family communication, the negative impacts have become more prominent [3,11,12]. Factors such as restricted freedom, changes in nutrition, sleep, and daily habits, decreased interpersonal interactions, increased screen time, the prevalence of negative news about the pandemic in the media, and fear of death have adversely affected family relationships [3,11,12]. Additionally, changes in parents' work lives, economic hardships, job loss, and heightened stress and anxiety during the pandemic have made it more challenging for them to cope, leading to negative parenting experiences [11,12]. In a study conducted by Amin et al., an anxiety scale was applied to adults during the pre-quarantine and post-quarantine periods, revealing a statistically significant increase in anxiety levels among individuals with moderate anxiety during the quarantine period [13]. The results of our study suggest that the low attachment scores observed in children who contracted COVID-19 may be attributed to these negative parenting experiences, potentially affecting the parents' attachment to their children as well.

In a study conducted by Karakaş et al., the attachment of mothers who experienced physical or sexual abuse during childhood to their children was examined. The findings indicated no difference in secure attachment between the children of abused and non-abused mothers, although the attachment scores of the mothers to their own parents were significantly lower [14]. The study did not demonstrate intergenerational transmission; however, it noted that this transmission was interrupted by the mothers' higher education levels [14]. In our study, we were unable to assess the educational levels of families, but we hypothesize that negative parenting experiences can adversely affect attachment in adolescents, both physically and psychosocially.

Research has shown that increased stress and constant close contact can serve as risk factors for violence and aggressive behaviors [6,11]. During the COVID-19 pandemic, there has been a reported increase in the number of children experiencing maltreatment and abuse [6,10]. This situation negatively impacts family bonds [6,8,11,12]. Our study found that, despite no loss within the family, adolescents who contracted COVID-19 had particularly low attachment scores to their fathers. This effect may be explained by potentially weak past family bonds, fear of death, isolation, or negative parenting experiences that could lead to family violence, poor communication between parents, and low parent-child communication. Additionally, negative family attitudes and relationships might undermine adolescents' personal hygiene, protection, and health practices, which could directly adversely affect their health.

Yang et al. conducted a study in China involving 442 families, finding that adolescents with secure attachments to their parents exhibited lower levels of COVID-19-related anxiety and depression [15]. Similarly, Zawilska et al. investigated the effects of the COVID-19 pandemic on adolescents, reporting an increase in depression and anxiety compared to the pre-pandemic period [16]. Close et al. also reported an increase in emergency department visits due to depression and suicide attempts among adolescents following the pandemic [8].

An increase in depressive symptoms in children is thought to negatively affect parental attachment [9]. Although we cannot assess pre-pandemic attachment levels, our study suggests that the depression and anxiety experienced during isolation negatively impacted parental attachment.

Orgiles et al. reported increased restlessness, irritability, anger, and screen exposure among children aged 3-18 during the pandemic [12]. The increased time spent alone and the heightened screen exposure may negatively affect parental attachment [8,9,12].

Adolescence is a transitional period from childhood to adulthood, characterized by rapid changes in physical appearance as well as physiological, psychological, and social functioning [17]. During this time, adolescents experience advancements in their mental and emotional skills, and their autonomy increases [17,18]. Consequently, they begin to seek new activities, undertake new challenges, and spend more time with peers [17,18]. In adolescence, the primary attachment figures are typically the parents [18]. However, unlike in infancy, adolescents tend to interact more with friends and peers rather than with their parents or caregivers, leading to a distancing from these attachment figures [18]. Therefore, parental attachment during adolescence may evolve into different forms [17,18].

In our study, we focused on adolescents with an average age of 10 years and four months. At this age, peer communication plays a more significant role than family relationships [3,12,17,18]. However, the COVID-19 pandemic, which resulted in school closures, negatively impacted peer interactions. Children who were temporarily removed from institutional educational environments and peer social contact were deprived of adequate cognitive, emotional, and physical stimuli appropriate for their age [3,12]. A study conducted during the COVID-19 pandemic reported that adolescents who experienced social distancing from peers suffered negative effects on their secure attachment to parents [19]. Given that our study observed negative impacts on peer communication during isolation, we believe this also adversely affected parental attachment.

Our study evaluated parental attachment scores by gender and found no significant differences between the two. According to classical attachment theory, the fundamental survival instincts are the same for both male and female infants; thus, attachment is considered independent of gender [20]. Therefore, we did not observe any gender differences in our findings.

A positive and significant correlation was identified between maternal and paternal attachment scores in our study. In other words, children who exhibited a more secure attachment to their mothers also tended to have a more secure attachment to their fathers. The literature indicates that many factors independently influence maternal and paternal attachment [21]. However, there is currently no study that considers both maternal and paternal attachment together. Our research suggests that this positive correlation between maternal and paternal attachment may arise from healthy family communication.

Strong attachments to parental figures and positive attachment experiences are known to be associated with a more comfortable future, increased resources for coping with stress, and higher self-esteem [18,22]. Therefore, attachment style plays a crucial role in adolescents' future functionality and psychosocial development.

In our study, we hypothesize that during the COVID-19 pandemic, factors such as increased solitary time, heightened screen exposure, social withdrawal from peers, fear of death, elevated parental stress, and negative childhood or parenting experiences negatively impacted child-parent attachment [13-15]. Additionally, it can be speculated that adolescents with lower pre-pandemic parental attachment may exhibit lower adherence to self-care and hygiene rules, potentially leading to a higher incidence of COVID-19 infections among these adolescents. Further research is needed to explore this issue.

Limitations

There are also limitations to our study. It was conducted after the COVID-19 isolation periods, and we do not know the parental attachment status of the patients prior to the COVID-19 pandemic. Therefore, it is not yet possible to make a direct comparison between pre- and post-COVID-19 periods. However, the negative effects on child-parent bonding during the COVID-19 period are evident. Additionally, our study was based on data collected from a single university hospital in our country, which may not represent the entire nation. Thus, multicenter studies are needed to validate these findings.

## Conclusions

The COVID-19 pandemic has brought many negative experiences for children as well as adults. We think that situations such as the isolation of children from their social environment and peers, the increase in the time they spend alone at home, the fear of death due to the COVID-19 infection, and negative family experiences due to increased stress in the family have also negatively affected parental attachment. As is known, insecure attachment is associated with negative outcomes such as anxiety, depression, academic failure, and internalization problems in adulthood. In order to prevent these negative consequences, it will be important to understand the importance of family relationships and social environment during adolescence and to make appropriate interventions and lifestyle changes.
